# Toward a Metagenomic Understanding on the Bacterial Composition and Resistome in Hong Kong Banknotes

**DOI:** 10.3389/fmicb.2017.00632

**Published:** 2017-04-13

**Authors:** Yoshitaro Heshiki, Thrimendra Dissanayake, Tingting Zheng, Kang Kang, Ni Yueqiong, Zeling Xu, Chinmoy Sarkar, Patrick C. Y. Woo, Billy K. C. Chow, David Baker, Aixin Yan, Christopher J. Webster, Gianni Panagiotou, Jun Li

**Affiliations:** ^1^Systems Biology and Bioinformatics Group, School of Biological Sciences, Faculty of Sciences, University of Hong KongHong Kong, China; ^2^School of Biological Sciences, Faculty of Science, University of Hong KongHong Kong, China; ^3^Healthy High Density Cities Lab, HKUrbanLab, University of Hong KongHong Kong, China; ^4^Department of Microbiology, University of Hong KongHong Kong, Hong Kong; ^5^State Key Laboratory of Emerging Infectious Diseases, University of Hong KongHong Kong, Hong Kong; ^6^Research Centre of Infection and Immunology, University of Hong KongHong Kong, Hong Kong; ^7^The Carol Yu Centre for Infection, University of Hong KongHong Kong; ^8^Guangzhou Center for Disease Control and PreventionGuangzhou, China; ^9^Department of Systems Biology and Bioinformatics, Leibniz Institute for Natural Product Research and Infection Biology, Hans Knöll InstituteJena, Germany

**Keywords:** metagenomics, antibiotics resistance, dissemination potential, horizontal gene transfer

## Abstract

Currency is possibly one of the main media transmitting pathogens and drug resistance due to its wide circulation in daily life. In this study, we made a comprehensive characterization of the bacterial community present on banknotes collected from different geographical regions of Hong Kong (HK) by performing *in vitro* characterization of the bacterial presence and resistome profile, as well as metagenomic analysis including microbial diversity, the prevalence of potential pathogens, the dissemination potential of antibiotic-resistance genes (ARGs), among others. When comparing the bacterial community of HK banknotes with other HK environmental samples, including water and marine sediment, we revealed that HK banknotes cover nearly 50% of total genera found in all the environmental samples, implying that banknotes harbor diverse bacteria originated from a variety of environments. Furthermore, the banknotes have higher abundance of potential pathogenic species (~5 times more) and ARGs (~5 times more) with higher dissemination potential (~48 times more) compared with other environmental samples. These findings unveiled the capabilities of this common medium of exchange to accommodate various bacteria, and transmit pathogens and antibiotic resistance. Furthermore, the observed independence of microbiome profile from the city's topological indices led us to formulate a hypothesis that due to their high circulation banknotes may harbor a homogenized microbiome.

## Introduction

Antibiotic resistance has become a global public health concern in the past decades. Bacteria with antibiotic resistance including methicillin-resistant *Staphylococcus aureus* (MRSA) and multi-drug-resistant *Mycobacterium tuberculosis* (MDR-TB) have been observed around the world (Bassetti et al., 2009; Raviglione and Sulis, 2016). Moreover, drug resistance in parasites/viruses such as malaria, HIV, and influenza has been also reported (Richman et al., 1994; Fairhurst and Dondorp, 2016; Li J. et al., 2016). The emergence of these resistant microorganisms has been accelerated by overusing of antibiotics on both humans (Llor and Bjerrum, 2014) and livestock (Landers et al., 2012). Furthermore, the rapid dissemination of such antibiotic resistance poses another threat for spreading the resistance across different opportunistic microbes worldwide (Molton et al., 2013). Therefore, investigating the reservoir of the antibiotic resistance and pathogenic factors in vehicles of interaction between humans and the microbial world has become an important task.

Money, consisting of currency notes and coins, is a circulating medium of exchange and a measure of values in markets. Due to its frequent circulation in daily life, money could get easily contaminated. The contamination source of microbes on currency can be food, water, air, soil, dust, or handler, etc. (Awe et al., 2010). Especially, human pathogens can be transmitted to money due to the personal unhygienic habits, e.g., touching currency after coughing, sneezing or handling food (Ahmed et al., 2010), or routine money handling practice of cashier or salesman (Badvi et al., 2013). Previous studies have revealed that 70–94% of banknotes and coins harbor various bacteria and viruses on the surface in different nations such as the United States, China, India, etc. (Sharma and Sumbali, 2014). It is also verified by laboratory simulations that bacteria and viruses can survive on the surface of banknotes or coins for 1–13 days (Kramer et al., 2006). Furthermore, the transmission of pathogenic species, such as *Escherichia coli* or *S. aureus*, from banknotes to humans has been observed (Gedik et al., 2013). Additionally, bacteria with antibiotic resistance on currency notes have been reported around the world (Gabriel et al., 2013; Akoachere et al., 2014; Kumar et al., 2015). It was suggested that “banknotes could serve as a vehicle for transmission of drug resistant pathogenic” (Akoachere et al., 2014; Angelakis et al., 2014). Moreover, the banknotes collected from unique places such as hospitals are considered to be contaminated by pathogens such as *S. aureus* (Angelakis et al., 2014).

The research methods for exploring microbial communities on currency notes were previously dominated by culture-dependent experiments and the 16S rRNA gene sequencing. Studies using culture-dependent experiments were limited to assess the presence of particular culturable bacteria (Kuria et al., 2009; Moosavy et al., 2013). Although the 16S rRNA gene sequencing has overcome the problems of investigating culturable bacteria by identifying all bacteria present in an environment exhaustively (Pereira da Fonseca et al., 2015), it still suffers from the problem of low resolution of taxonomic profiling and lack of functional description for bacterial communities. In contrast, the shotgun sequencing enables biologists to broaden their interests to the functional perspective by analyzing the whole DNA fragments from an ecological environment. Jalali et al. recently applied for the first time a metagenomic approach and identified pathogens including *S. aureus, Corynebacterium glutamicum* and various antibiotic-resistance genes (ARGs) in Indian paper currency notes collected in New Delhi (Jalali et al., 2015). However, the sample size was not enough to draw a concrete statistical conclusion in the study. In addition, the authors did not investigate whether these bacteria are indeed active or the dissemination potential of ARGs.

Several reasons that include interconnected biological, ecological, social and technological processes have put South East Asia at risk for infectious diseases with pandemic potential. Hong Kong (HK) is a cosmopolitan city and due to its high population density and high mobility of people, combined with the hot-humid climate, it continually bears the high risk of the spread of infectious diseases. In fact, HK has experienced several large-scale outbreaks of infectious diseases in the last decades, i.e., flu pandemic in 1968 (Cockburn et al., 1969), an outbreak of avian flu in 1997 (Chan, 2002), SARS epidemic in 2003 (Hung, 2003), swine flu pandemic in 2009 (Wu et al., 2010). Therefore, it is of clinical importance to examine whether HK banknotes could serve as pathogen reservoirs and vehicles through which pathogenic bacteria and infectious diseases could be transmitted to humans. Research in this direction includes the development of novel tools and methodologies to assess the bacterial pathogenicity profile and antibiotic-resistance distribution in highly populated cities. In this study, we have assessed the bacterial community composition, the prevalence of pathogenic bacteria, the abundance and dissemination potential of ARGs of HK banknotes, and evaluated the commonalities and differences of the taxonomic and functional characteristics of the bacterial community between banknotes and other local environmental samples. Lastly, we integrated our biological data with different city network metrics, such as connectivity analysis, population densities, and physical locations, to investigate the influence of anthropogenic factors on the microbiome that we are exposed through the daily handling of banknotes.

## Results

### Microbial community on HK banknotes

**Figure 1:**
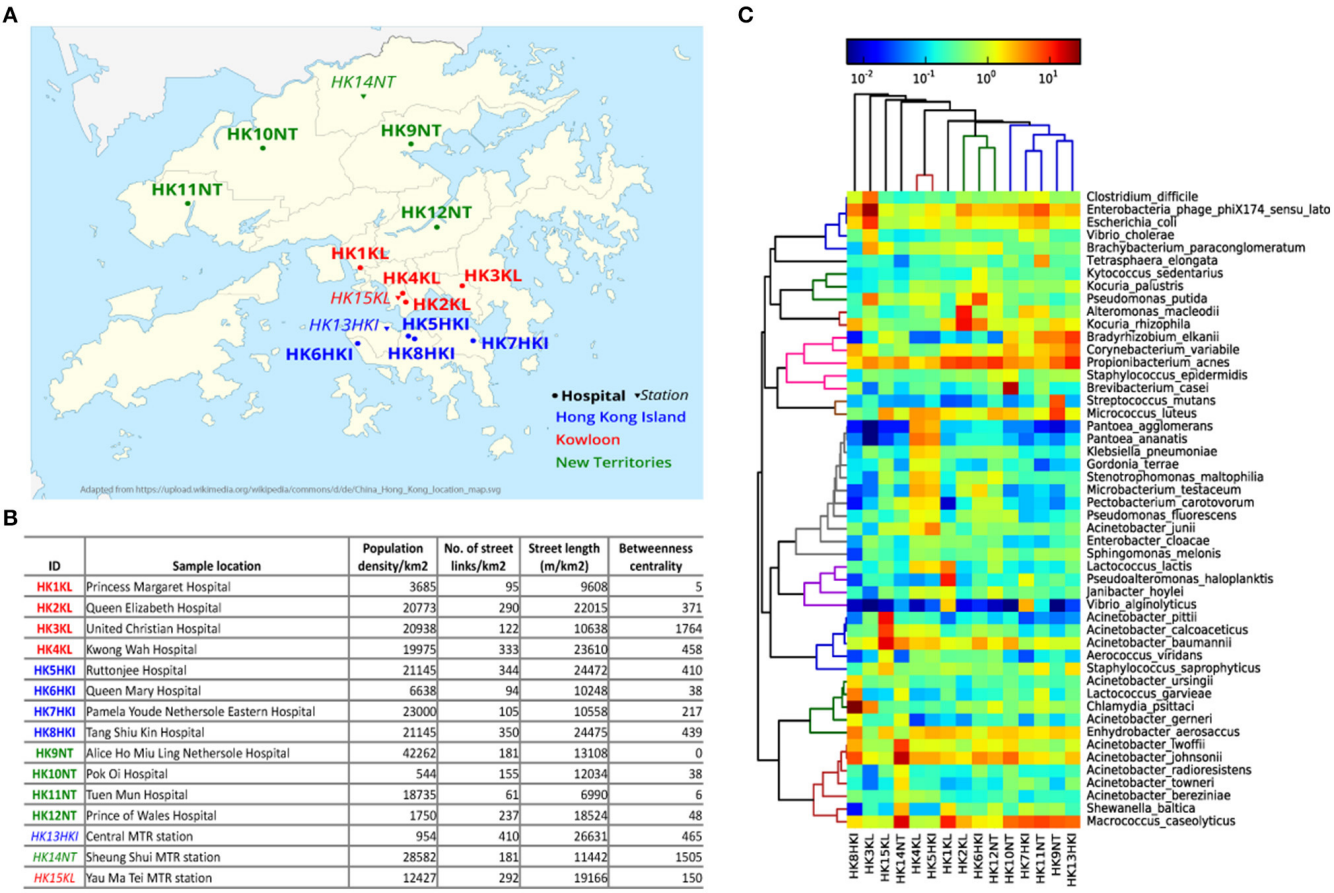
**Microbial community on HK banknotes. (A)** Sampling locations (See Table S1 for details). The hospitals are marked with circles and bold font, while metro stations were marked with triangle and italic font. Three different colors: blue, red, green denotes different regions in HK: HK Island, Kowloon, and New Territories, respectively. **(B)** City metrics of sampling locations of HK banknote samples. The betweenness centrality is measured by the mean of all street links within a 50 m-catchment radius at 300 m scale. **(C)** Heat map of top 50 abundant species. The colors in hierarchical clustering tree refer group information.

HK $20 notes were collected from cashiers at 12 hospitals and three metro stations located in three geographical regions of HK: HK Island (HKI), Kowloon (KL), New Territories (NT; Figure 1A). Five sampling locations (four hospitals and one metro station) for each geographical region of HK were chosen. The variability in most of the city's network metrics is high among the different sampling locations (Figure 1B). For example, the representative population density of sample HK9NT was 77 times higher than that of sample HK10NT even though both sampling locations belong to the same region (NT). The number of street links (links/km2) was almost seven times higher in sample HK13HKI than sample HK11NT.

To examine whether microbes can actually survive on the banknotes, we attempted to cultivate the extracted cells from banknotes in different media, including LB agar and four different selective media [Tryptic soy agar (TSA), chocolate agar, bile esculin agar (BEA) and phenylethyl alcohol agar (PEA; Table S1]. The colonies observed on the cultivation plates (Figure S1) indicate that banknotes potentially offer the environment to harbor living bacteria. In order to evaluate experimentally the antibiotic resistant levels of bacteria found on the HK banknotes, we measured the minimum inhibitory concentration (MIC) for selected antibiotics (ampicillin, chloramphenicol, ciprofloxacin, erythromycin, kanamycin, spectinomycin, tetracycline). Any antibiotics did not have significant differences on the comparison between the samples from hospitals and the ones from stations. For example, regarding ampicillin and spectinomycin, there was no significant difference (*p* = 0.2357 and *p* = 0.2816, respectively, Wilcoxon rank sum test; Table S2).

All the 15 samples were processed for metagenomic sequencing generating 98.5 Gbp of sequencing data (average 6.57 Gbp per sample). We adopted a homology search method (Huson et al., 2011) to quantify the taxonomic profile at different level including species of the microbes on banknotes. Overall, 22% of quality-controlled reads were mapped to bacteria, 13% to eukaryote, 0.6% to viruses, and 0.04% to archaea, with the rest 64.36% of sequenced reads mapped to unknown origin. All the bacteria identified in the cultivation-based experiments with selective media (see above) were discovered in the taxonomic profile of the banknote samples (Table S1). The most abundant bacteria genus was *Acinetobacter* (16.27%), where some potentially pathogenic species of *Acinetobacter* (*A. baumannii, A. lwoffii, A. calcoaceticus, A. junii*) were in relatively high abundance in the HK banknotes. However, at species-level, *Propionibacterium acnes* was the most abundant (4.9% on average), and the most abundant strain of *P. acnes* was *P. acnes C1* (28.9% on average), which was isolated from a sarcoidosis patient (Minegishi et al., 2013). Besides the *C1* strain, five other pathogenic strains (*KPA171202, ATCC11828*, SK187, *HL099PA1, SK182B-JCVI*) were identified among the detected 31 strains (Figure S2). The total relative abundance of these potential pathogenic species was on average 35.6% of the total bacteria among the 15 samples. Moreover, widely recognized potential pathogens such as *E. coli* (1.96% on average), *Clostridium difficile* (0.88% on average), and *Vibrio cholerae* (0.48% on average) were detected as relatively abundant species on the HK banknotes (Figure 1C). Even though we attempted to analyze also these species at the strain level using PanPhlAn (Scholz et al., 2016) and ConStrains (Luo et al., 2015), the low coverage could not allow us reaching in more robust conclusions on possible pathogenicity. We subsequently investigated the overall compositional differences (beta-diversity) among the banknotes samples and the possible influence of geographical regions (HKI, KL, NT) and facilities (hospitals, stations) on the bacterial community. Comparison of species composition in different sample groups indicates that there is no significant difference between different regions (*p* = 0.7418, ANOSIM). Furthermore, to investigate whether the city's topological indices may have an impact on the microbiome of the HK banknotes, we carried out Spearman linear correlation analysis between species diversity and abundance, and the anthropogenic indices such as population density, street links density, street length, and network centrality (betweenness) in certain scales (see Figure 1B and Section Materials and Methods for definition). Out of such 51 tests, the correlations between beta-diversity (Weighted UniFrac distances) and the betweenness centrality in small-scale networks showed statistical significance (*p* = 0.0297 at 300 m scale, ADONIS; Figure S3), but none remained significant after multiple test correction. This observed independence of the banknotes' microbiome from the characteristics of the sampling locations led us to formulate a hypothesis that due to their high circulation banknotes may harbor homogenized microbiome, which serves as a kind of fingerprint of the city's microbiome (Figure S4). However, much more intensive sampling is needed to confirm whether this pattern of homogenization could be confirmed.

### 16S rRNA-based comparison of HK banknotes and other environmental samples

**Figure 2:**
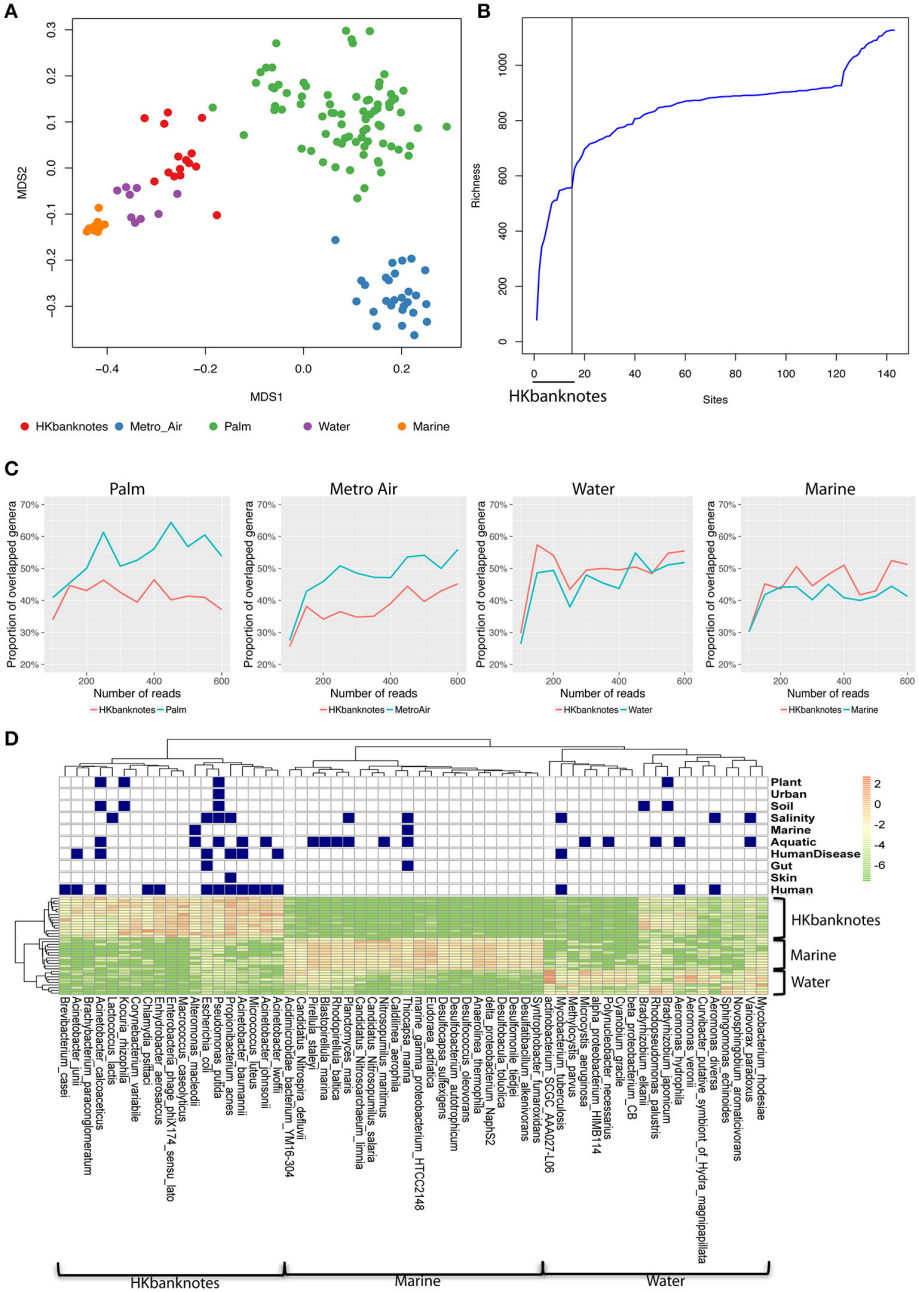
**16S rRNA-based comparison of HK banknotes and other local environments. (A)** MDS plot for taxa comparison at 16S rRNA level using Bray-Curtis distance based on 43 genera with mean abundance > 0.5%. The HK banknotes (red color) were distinct from other environmental samples (*p* = 0.0001, ANOSIM). **(B)** Genera accumulation curve. The first 15 samples are the HK banknotes followed by the MTR air, palms, marine sediments, and water samples. **(C)** Proportion of overlapped genera. **(D)** Heat map of top 20 abundant species of HK banknotes, water, and marine samples. Navy color blocks indicate that the species can be adapted to the corresponding environment.

To highlight the unique characteristics of HK banknotes, we performed a comparison with other HK environmental samples collected from residents' palms (Wilkins et al., 2016), air in metro stations (Leung et al., 2014), drinking water, and marine sediments (Guo et al., 2016) in terms of taxonomic composition at genus-level based on 16S rRNA gene sequencing. The results indicate that the overall taxonomic composition of banknotes is significantly different from other environmental samples (*p* = 0.0001, ANOSIM; Figure 2A). We further identified the genera with significantly different abundance between the HK banknotes and other environmental samples. We found 18 genera including *Vibrio, Pseudomonas, Paracoccus, Haemophilus, Bradyrhizobium, Acinetobacter, Corynebacterium, and Enterobacter* that were enriched in the HK banknote samples (*p* < 0.05, *t*-test). These genera are generally found in a large variety of environments such as water, soil, animals, and humans (Bernard, 2012; Jeong et al., 2013; Miquel et al., 2013; Chen et al., 2014; Takemura et al., 2014; Cardines et al., 2015; VanInsberghe et al., 2015).

We also observed that the HK banknotes harbor more diverse bacteria compared with other HK environmental samples. The accumulation curve of the genera richness among different environmental samples reveals that the HK banknotes contribute around half of the total genera in all HK environmental samples (Figure 2B, Figure S5). Furthermore, we noticed that around 60% of identifiable genera from the HK banknotes were unique compared with the palm and MTR air samples; nearly half of the genera found on the HK banknotes were unique compared to the water and marine sediment samples (Figure 2C). This observation implies that the HK banknotes offer an environmental niche with the capacity to harbor various bacteria originated from different habitats. The most abundant species on the HK banknotes were human-related, but many species could be also categorized to multiple environments (Figure 2D) including soil, aquatic, and salinity, etc. In contrast, the water and marine sediment samples contain species that can exist in the limited environment. These results imply that the HK banknotes harbor various types of bacteria from different environmental niches and provide one more piece of evidence that, due to their high circulation, banknotes may be a good representation of the most prominent members of a city's microbiome.

### Taxonomic and functional comparison of banknotes and other environmental samples based on shotgun metagenomes

**Figure 3:**
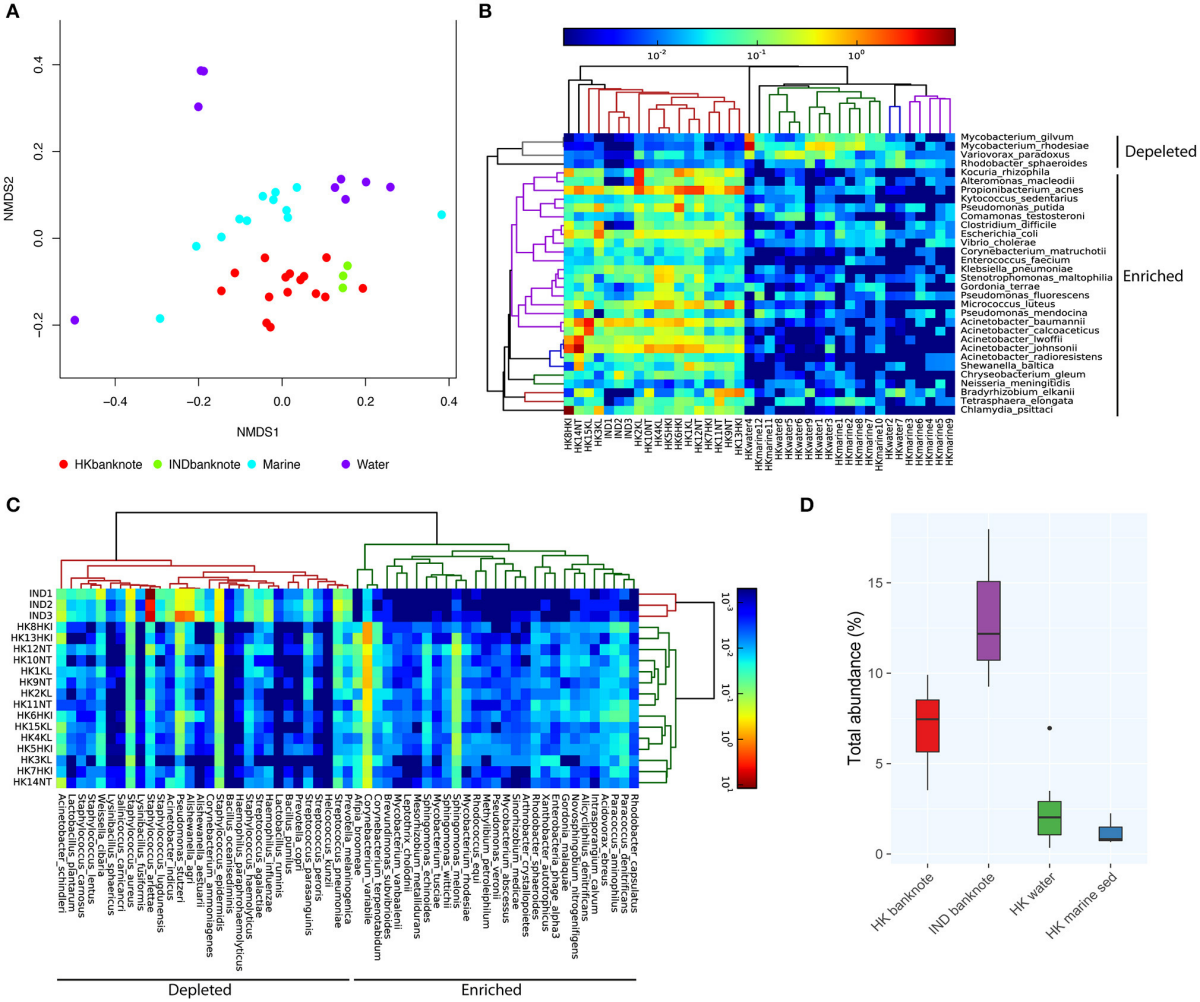
**Shotgun metagenomic-based comparison of banknotes (HK and Indian) and other HK environmental samples. (A)** NMDS plot based on weighted UniFrac distance at species-level using high abundant species among the samples. The difference between the HK and Indian banknote samples is insignificant (*p* = 0.0773, ANOSIM). The banknote samples formed a cluster, which is significantly different from the water and marine sediment samples (*p* = 0.0001, ANOSIM). **(B)** Heat map of 29 HK-enriched species and top 30 of Indian-enriched species. **(C)** Heat map of differentially abundant species between banknotes and other environments. **(D)** Comparison of total abundance of potential pathogenic species.

To investigate in higher resolution the taxonomic composition and functionality of microbes present in banknotes compared to other environmental samples, we retrieved publicly available shotgun metagenomic sequencing data from Indian banknotes, HK water samples, and HK marine sediment samples. The non-metric multidimensional scaling (NMDS) based on species-level weighted UniFrac distances revealed that the difference between the HK and Indian banknote samples is insignificant (*p* = 0.0773, ANOSIM; Figure 3A), while a more thorough investigation of the commonalities and differences of these two groups is given in the next section. The banknote samples formed a cluster, which is significantly different from the water and marine sediment samples (*p* = 0.0001, ANOSIM; Figure 3A). More specifically, 28 species (*E. coli, Pseudomonas putida, V. cholerae, P. acnes*, etc.) were enriched while four species were depleted in the banknotes, including *Rhodobacter sphaeroides, Variovorax paradoxus, Mycobacterium gilvum, Mycobacterium rhodesiae* when compared with the water and marine sediment samples (Figure 3B; *p* < 0.05, *t*-test). Four commonly existing species in both HK and Indian banknotes were identified (*t*-test, *p* > 0.7); three *Acinetobacter* species (*A. baumannii, A. pittii, A. calcoaceticus*) and *Klebsiella pneumoniae*. Besides the four species, *Vibrio cholera* was also identified commonly in both groups of banknotes (*p* = 0.693, *t*-test). We further investigated the abundance of potential pathogens and the result shows that the potential pathogenic species are in higher abundance (4.76 times more) in banknotes than other environmental samples (*p* = 9.6e-10, Wilcoxon rank sum test; Figure 3D). When comparing the potential pathogenic species with significantly different (*p* < 0.05, *T*-test) abundance between the banknotes and other samples, we identified 110 potentially pathogenic species such as *E. coli, V. cholera*, and *Salmonella enterica*, among others.

**Figure 4:**
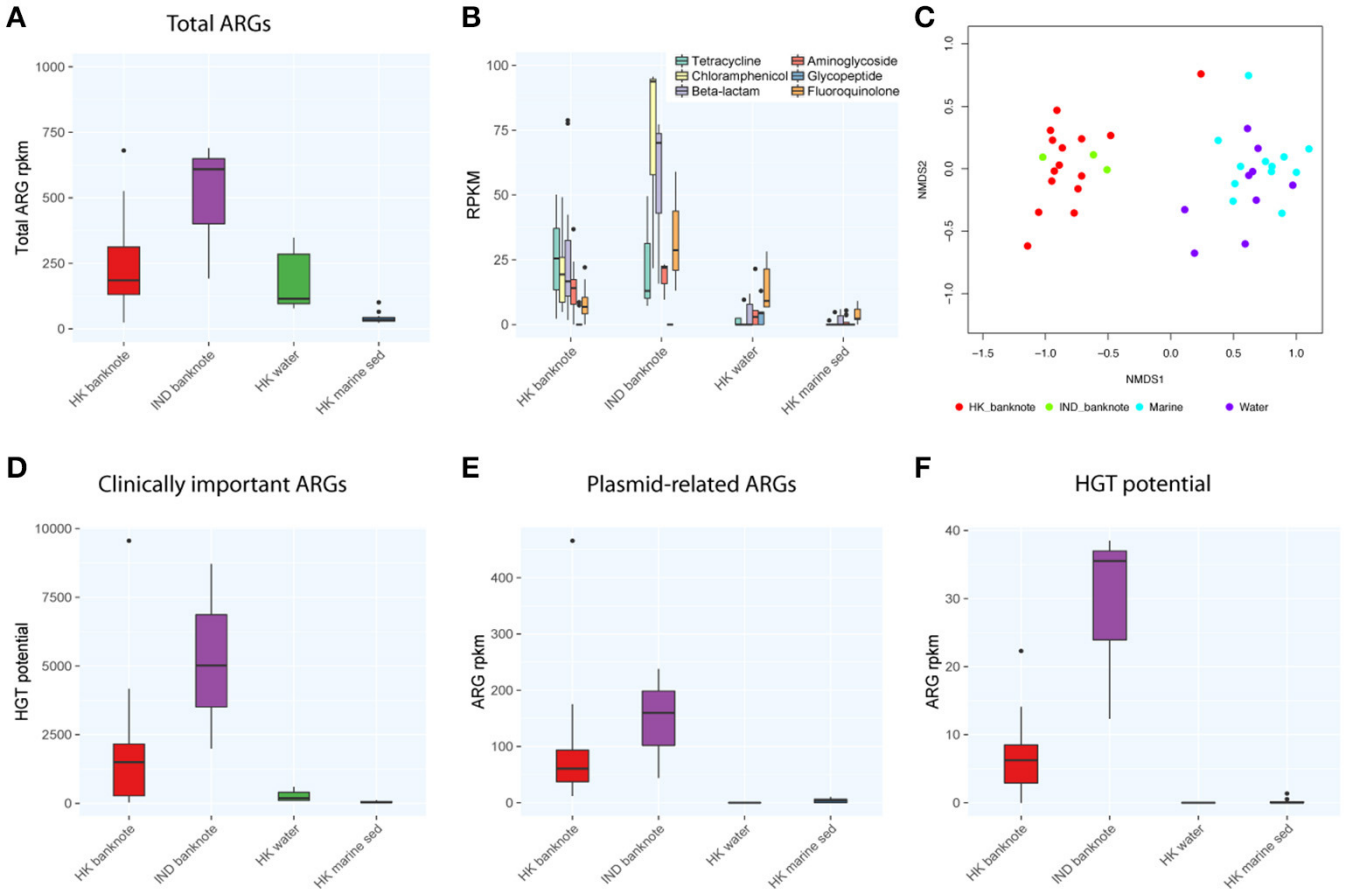
**Comparison of abundance and dissemination potential of antibiotic resistant genes (ARGs) among HK banknotes, Indian banknotes, HK water, and HK marine sediments. (A)** Comparison of total abundance of ARGs. **(B)** Comparison of total abundance of categorized ARGs. **(C)** NMDS plot based on the composition of ARG families. **(D)** Comparison of total abundance of clinically important ARGs. **(E)** Comparison of total abundance of plasmid-related ARGs. **(F)** Comparison of total HGT potential.

We also compared the total abundance of ARGs between banknotes and other environmental samples. Our result indicates that the banknotes samples have significantly higher total ARG abundance (4.86 times more) than other environmental samples (*p* = 6.1e-05, Wilcoxon rank sum test; Figure 4A). However, experimental validations such as qPCR quantification are required to confirm this observation, since the incompleteness of the ARG databases possibly biased the observation. The detected ARGs could be further classified into six groups encoding resistance against different antibiotics: tetracycline, chloramphenicol, beta-lactams, aminoglycoside, glycopeptide, and fluoroquinolone. We found that chloramphenicol-resistance genes were the most abundant ARGs (9.42% on average) on the banknotes followed by tetracycline (9.13% on average) and beta-lactams (8.02% on average; Figure 4B). In contrast, fluoroquinolone-resistance genes were most abundant in water (8.20% on average) and marine sediment samples (9.55% on average). Further analysis of dissimilarity between sample groups in terms of the ARGs abundance revealed that the ARGs compositional difference between the two-banknote groups was insignificant (*p* = 0.5039, ANOSIM) while the ARGs compositions of banknotes samples were significantly different with the HK water and marine sediment samples (*p* = 0.0001, ANOSIM; Figure 4C). Besides, we found that the total abundance of clinically important ARGs (see Section Materials and Methods for definition) in the banknotes was significantly higher than other environmental samples (*p* = 2.4e-07, Wilcoxon rank sum test; Figure 4D). The *tetK* gene was the most abundant clinically important ARG on the banknotes, whereas, three out of twelve (25%) marine sediment samples contain either *ermB* or *tetM* genes. Furthermore, we evaluated the dissemination potential [total abundance of plasmid-related ARGs and horizontal gene transfer (HGT) potential] of the identified ARGs. The banknotes have a significantly higher abundance (47.80 times more) of plasmid-related ARGs (*p* = 1.0e-07, Wilcoxon rank sum test) and significantly higher HGT potential (16.50 times more; *p* = 2.2e-16, Wilcoxon rank sum test) than other environmental samples (Figures 4E,F). The concomitant identification of clinically important ARGs with high dissemination potential suggests that currency could possibly pose a health risk.

In addition, we analyzed the biosynthetic gene clusters (BGCs) to identify physically clustered gene sets responsible for the synthesis of microbial secondary metabolites. Interestingly, the BGCs of thiopeptide (*p* = 0.001, *t*-test) and oligosaccharide (*p* = 0.001, *t*-test) were significantly enriched in the banknotes compared to the other environmental samples (Figure S6). Thiopeptide is a sulfur-rich, highly modified heterocyclic peptide antibiotic drug, and oligosaccharide is known as a substrate for some bacteria to produce short-chain fatty acids.

### A comparative analysis of HK and Indian banknotes

We subsequently investigated the differentially abundant species between the HK and Indian banknotes. We found that 29 species, including *P. acnes, P. avidum*, and *Sphingomonas melonis*, are significantly more abundant in the HK banknotes (Figure 3C). *P. acnes* was the most abundant species in the HK banknote samples on average, as mentioned above. *P. avidum* has a pathogenic potential and the ability to cause clinically relevant infections (Wildeman et al., 2016). *S. melonis* has been reported as a novel pathogen that causes brown spot on yellow Spanish melon fruits (Buonaurio et al., 2002). On the other hand, Indian banknotes had 216 species significantly more abundant such as *Staphylococcus arlettae, Staphylococcus hominis, Aerococcus viridans, Pseudomonas stutzeri, Alishewanella agri*, etc. (*p* < 0.05, *T*-test).

When comparing the total abundance of potential pathogens of banknotes from the two countries, we found that the HK banknotes have significantly less abundance of potential pathogens compared with the Indian banknotes (*p* = 0.0049, Wilcoxon rank sum test; Figure 3D). Regarding the number of potential pathogenic species, there is no significant difference between the two groups (the HK banknote samples harbor 164 potential pathogens while Indian banknote samples contain 170). The most abundant potential pathogen among the HK banknotes was *P. acnes* (4.90% on average), which is a dominant skin commensal and opportunistic pathogen as well. Moreover, other pathogenic species of *Acinetobacter* (*A. baumannii, A. lwoffii, A. calcoaceticus, A. junii*) were in relatively high abundance in the HK banknotes (Figure 1C). Additionally, *C. difficile* (0.88% on average) was also abundant in the HK banknote samples. *C. difficile* is widely known due to *C. difficile* infection (CDI), which causes from diarrhea to severe inflammation in colon. The spores of *C. difficile* are resistant to heat, acid, and antibiotics, so they can survive in the environment for months and be easily transmitted (Martinez et al., 2012).

To evaluate the difference of ARGs between the HK and Indian banknotes, we compared the total abundance of ARGs of the two groups. The results showed that the HK banknotes had less total abundance of ARGs than the Indian banknotes, but the difference was insignificant (*p* = 0.4265, Wilcoxon rank sum test; Figure 4A). Additionally, the HK banknotes have significantly less abundance of clinically important ARGs (*p* = 0.0098, Wilcoxon rank sum test; Figure 4D). The *tetK* and *msrA* genes were the most abundant clinically important ARGs on the HK and Indian banknotes, respectively. Regarding the dissemination potential in ARGs, the HK banknotes have less abundance of plasmid related ARGs than Indian, but the difference was insignificant (*p* = 0.3603, Wilcoxon rank sum test; Figure 4E). However, the HK banknotes have significantly less HGT potential in ARGs than the Indian (*p* = 1.5e-07, Wilcoxon rank sum test; Figure 4F). On average, “aminoglycoside phosphotransferase (APH3)” was the ARG family that had the highest HGT potential on both the HK and Indian banknotes.

In addition, we analyzed the BGCs to identify physically clustered gene sets responsible for the synthesis of microbial secondary metabolites. Interestingly, the BGCs of thiopeptide (*p* = 0.031, *t*-test) and bacteriocins (*p* = 0.024, *t*-test) were significantly enriched in the HK banknotes compared to the Indian banknotes (Figure S6), and the biosynthetic gene abundances of these two metabolites have a weak positive correlation (*p* = 0.059, Spearman). Thiopeptide are sulfur-rich, highly modified heterocyclic peptide antibiotics, and bacteriocins restrict the activity to strains of species related to the producing species and particularly to strains of the same species. In addition, the abundance of thiopeptide synthesis genes has a strong positive correlation with the total abundance of skin-related species.

## Discussion

To establish roadmaps for discovering how microbes travel between different parts of our body and between various environments we come into contact every day, the development of tools and techniques for elucidating microbial interaction has attracted a lot of attention (Bikel et al., 2015). Such knowledge has a potential for predicting the emergence of diseases linked to an altered microbiome in our body, and how the design of our city and biodiversity of our natural environment can benefit human health by mitigating epidemics and anti-microbial resistance (Kembel et al., 2012). Although currencies have been suggested as significant reservoirs and vectors in harboring and transmitting pathogens, ARGs and virulence factors, a rational sampling design has not implemented before. Here we embraced a proof-of-concept analysis of the bacterial network integrated with the city network for a better understanding on the existence, prevalence, and distribution of the virulence factors and ARGs of the microbes that we continuously carry.

In this study, we collected banknotes from hospitals and metro stations from different geographical regions of HK, we performed *in vitro* characterization of the particular bacterial presence and resistome profile as well as shotgun metagenomic sequencing. We made the following hypothesis: any specific location acts as a collection and distribution point, filtering and selecting, dampening, and amplifying trends in the natural ecology of microbes in a city as carried through trade networks. Hospitals, act as potent amplifiers and filters because they concentrate the coming together of people with higher than average pathogenic content in their personal microbial ecologies. Metro is one of the most frequent used public transportations for local populations; as such metro located stores represent another kind of microbial vector amplification, concentration and filtering node. Interestingly, our comparative analysis of bacterial composition, pathogenic content, and resistome profile in banknotes obtained from different regions, facilities and city network indices revealed no statistically significant differences. Furthermore, a comparison of HK banknotes with other local environmental samples indicates the higher bacterial diversity of the banknotes. Both findings offer some evidence that the high circulation of banknotes and their continuous exposure to the microbes that surrounds us may result to one of the most useful surveillance platforms for monitoring the “city's microbiome status.” Of course, we are fully aware that much more extensive and longitudinal sampling is required to further confirm our hypothesis.

The impact of the environment on the banknotes' bacterial composition/function was also shown when we included in the analysis banknotes from other countries (Figure S7). For example, the discovery of the higher abundance of marine bacteria in the HK banknotes than Indian banknotes (Figure S8) is due to the fact that HK is surrounded by the ocean while New Delhi, where the Indian samples were collected, is in central India. Another finding suggesting that banknotes reflect the city's particular characteristics is the high level of tetracycline resistance on the HK banknotes. It is consistent with two previous environmental studies in HK; Yang et al. showed that tetracycline-resistance gene was the most abundant in the wastewater treatment plants in HK by studying sludge samples using a metagenomic approach (Yang et al., 2013). Selvam et al. found that, in three HK rivers, the concentrations of tetracycline were relatively higher (highest in two rivers) among measured antibiotics (Selvam et al., 2016), which indicates the possibility that tetracycline resistance level is also abundant in the HK rivers. In addition, we observed that chloramphenicol resistance in Indian banknotes was the highest, and the total abundance of genes coding chloramphenicol acetyltransferase (CAT) was significantly higher than the HK banknotes. In fact, chloramphenicol was the first successful therapy of typhoid fever, which is the most common disease in India (Khandeparkar, 2010).

In the future, we plan to carry out a follow-up study by increasing the number of samples in a longitudinal study to investigate to what degree the currency can be used for monitoring the city's microbiome. Obviously, an exhaustive characterization of the temporal and spatial variation of the antimicrobial resistance and its integration with connectivity metrics indicating potential for though-movement at any point in the city network represents a novel methodological approach not only for mapping the landscape of risk associated with the currency circulation but for understanding the spreading dynamics in a future disease outbreak with direct implications in protecting public health.

## Materials and methods

### Sample collection, DNA extraction, and library preparation

HK dollars notes of $20 were collected from cashiers at twelve hospitals and three MTR stations located in three geographical regions of HK. The publish year of the collected currencies were ranged from 1998 to 2014. Both surfaces of banknotes per a sampling site were scraped with ESwab Collection & Transport System (BD company), which were pre-moisturized with 0.15 M NaCl and 0.1% Tween 20. PowerSoil DNA Isolation kit (MO BIO Laboratories) was used to extract microbial DNA following the official protocol. The extracted DNA were used to construct shotgun metagenomic libraries using the Nextera XT kit and following the standard protocol.

### Metagenomic sequencing and read quality control

The aforementioned DNA libraries were sequenced with Illumina HiSeq 1500 (101 bp PE) at the Centre for Genomic Sciences of The University of HK. The sequencing data of our samples were deposited in NCBI Sequence Read Archive (SRA) with accession number SRP101374. We have checked the negative control to make sure the potential laboratory contaminants do not bias the sequencing result by utilizing the reagents on a clean swab. The DNA concentration of the negative control was too low (0.0065 ng/uL) to create successful shotgun libraries. The sequenced reads were processed with a quality control step to remove the adapter regions and low quality reads, and mapped to human genome to filter out contaminations by following the previously described steps (Li X. et al., 2017). The sequencing data of other environmental samples that were used from comparison were obtained from NCBI SRA (accession number: SRR1293376, SRP033730, SRP061803), figshare (https://figshare.com/articles/Hong_Kong_indoor_air_project/1254033), and zenodo (https://doi.org/10.5281/zenodo.30279).

### Culture experiment and MIC measurement

Bacteria were scraped from the surface of banknotes (5X $20 per location) and collected into 500 μl phosphate-buffered saline (PBS) using ESwab Collection & Transport System (BD company). For culture experiments, each bacterial sample was then spread on five types of different agar plates [LB plate, Tryptic soy agar plate (TSA), Chocolate agar plate, Bile esculin agar plate (BEA) and Phenylethyl alcohol agar plate (PEA)] and plates were incubated at 37°C overnight. For MIC measurements, each bacterial sample was added into 5 ml MH broth and cultured overnight with 220-rpm agitation. Cell number of overnight culture was measured and then adjusted to 10^5^ per ml. Ninety-six well plate was used to measure MIC value for selected antibiotics (ampicillin, chloramphenicol, ciprofloxacin, erythromycin, kanamycin, spectinomycin, tetracycline) and the concentration of antibiotics was set from 1 to 200 μg/ml. Plates were incubated at 37°C for 16–20 h.

### Taxonomic assignment

The high-quality reads were mapped to nr database by DIAMOND (Buchfink et al., 2015) with the default setting. Lowest common ancestor (LCA) algorithm was implemented with LCA mapper from mtools of MEGAN5 (Huson et al., 2011) for taxonomy profiling of each read. The reads mapped to eukaryotes were removed for the further analysis. The relative abundance for each species was distilled from the LCA results using an *in-house* script. The bacterial species were queried against PATRIC (Wattam et al., 2014) for categorization. For potential pathogenic species annotation, three lists of potential pathogens (Kembel et al., 2012; Forsberg et al., 2014; Wattam et al., 2014) were combined. For 16S rRNA gene extraction, the filtered reads were mapped against ribosomal RNA SILVA reference sequences (SSURef_NR99_115; Quast et al., 2013) using assign_taxonomy.py from QIIME (Caporaso et al., 2010) with blast as the assignment method. Strain profiling of *P. acnes* was conducted according to the previously suggested reference-based approach (Oh et al., 2014).

### Calculation of alpha- and beta-diversity

Shannon alpha-diversity of each shotgun-sequencing sample was calculated with VEGAN (Dixon, 2009) based on the relative abundance of each species from 1 M subsampled reads. Weighted UniFrac distance was calculated PhylosEq (McMurdie and Holmes, 2013) based on the species-level taxonomic profile. To evaluate the community dissimilarities between 16S rRNA samples, Bray-Curtis dissimilarity was calculated based on the relative abundance of each genus. In addition, for the community dissimilarities of functional diversity was also assessed based on Bray-Curtis dissimilarity.

### Annotation of antibiotic-resistance genes (ARGs)

A million reads were randomly subsampled from each sample, and mapped to an in-house ARG database that is based on CARD (McArthur et al., 2013), ARDB (Liu and Pop, 2009), UniProt (UniProt Consortium, 2009) using blastx. The detected genes satisfied criteria (identity > 70% and coverage > 70%) were extracted and functionally annotated with Resfams (Gibson et al., 2015). The abundance of genes was calculated with RPKM (Reads Per Kilobase of transcript per Million mapped reads). The subsample reads were mapped to 27 resistance gene sequences as proposed previously for clinically important ARGs annotation (Munck et al., 2015).

### Estimation of dissemination potential of ARGs

All ARGs were mapped against NCBI plasmid RefSeq database (Pruitt et al., 2014) for estimating the plasmid-mediated HGT. The threshold of identity score was set to > 95% for further analysis. Using blastp (−e 1e-5), the protein sequences from all functional ARGs were mapped to the gene families that were acquired from the HGTree database (Jeong et al., 2016). The gene family of an ARG was defined with the gene family with the highest proportion of valid hits (1e-5 and coverage > 50% in query or subject). RANGER-DTL (Bansal et al., 2012) was used for phylogenetic reconciliation analysis based on the gene tree and species tree provided by the HGTtree. The HGT rate in each family was calculated by dividing the number of HGT events by the total phylogenetic tree length of that family.

### Annotation of biosynthetic gene clusters (BGC)

BGCs annotation were based on antiSMASH 3.0 (Weber et al., 2015) and modified as previously described (Donia et al., 2014; Ni et al., 2016). Briefly, the BGCs were identified and their abundances in each sample were calculated using pre-prepared identification of BGCs from the NCBI reference genomes under the following criteria: reads cover 50% of genes (after excluding the non-biosynthetic ones) in each BGC; the mean abundance of these genes were defined as the abundance of the identified BGC.

### Comparative statistical analysis

Statistical tests on comparisons of total abundance of potential pathogens, ARGs, clinically relevant ARGs, etc. were performed using Wilcoxon rank sum test on R. To detect significant items such as genera or species, analysis of similarities (ANOSIM) was implemented using QIIME (Caporaso et al., 2010). Also, adonis (QIIME) was employed to evaluate the significance of a variable in determining variation of distances.

### Measurement of urban morphometrics

We also examined the relationship between ecological distance between the microbial communities at the chosen sites (as measured by Bray-Curtis dissimilarity index and Shannon species diversity/heterogeneity index) and underlying urban connectivity, movement potential, and density of the sites in urban space. spatial Design Network Analysis (sDNA), a state-of- the-art urban network analysis technique (http://www.cardiff.ac.uk/sdna/) was employed to model multiple scale urban morphological metrics (morphometrics). The street network database of Hong Kong was subjected to sDNA analysis and the geographical connectivity and movement potential were measured by three morphometric indices: (i) through-movement potential or betweenness centrality of each street link was modeled at micro-meso-macro-level spatial scales (100, 200, 300, 400, 500, 600, 700, 800, 900, 1,000, 2,000, 3,000, 5,000, 7,500, 10,000, 15,000 m street catchments). These were subsequently aggregated for the street link “closest” to the study site as well as mean for all street links within a 50 and 100 m catchment radii and acted as proxy for multi-scalar urban movement density. (ii) The number and length of street segments per square Km within catchments of the study sites were measured through GIS queries. (iii) Poplulation density (in per sq. Km) for the local census geography (called Large Street Block Groups) containing each of the study sites was employed in the study as a conventional measure of urban density.

## Author contributions

JL and GP designed this study. YH and TD collected the banknote samples from sampling locations in HK. YH, KK, NY, and TZ analyzed the data. ZX performed the culture experiment and measured MIC. CS and CW measured anthropogenic indices of sampling locations in HK. YH, GP, and JL wrote the manuscript and PW, BC, DB, AY revised it.

### Conflict of interest statement

The authors declare that the research was conducted in the absence of any commercial or financial relationships that could be construed as a potential conflict of interest.
