# Supplementary material for: Toward a Metagenomic Understanding on the Bacterial Composition and Resistome in Hong Kong Banknotes
Source: Front Microbiol. 2017 Apr 13;8:632. doi: 10.3389/fmicb.2017.00632 (PMC5389987; doi:10.3389/fmicb.2017.00632)
Supplement: Supplementary file 1 [file DataSheet1.pdf]

Supplementary Materials

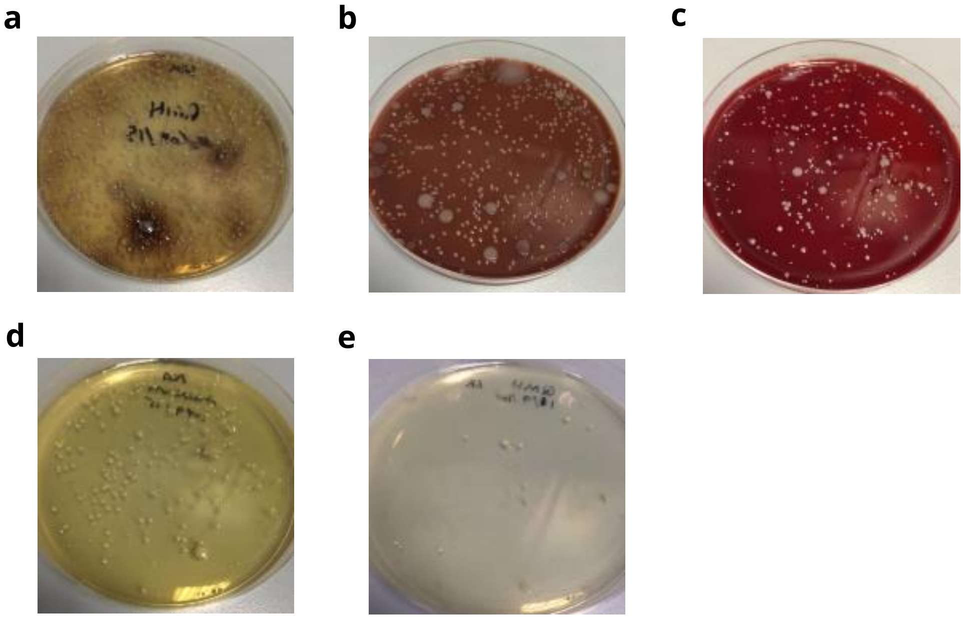

Figure S1. Colonies on selective media. (a) Bile esculin agar (BEA) (b) Chocolate agar (c) Phenyl ethyl alcohol agar (PEA) (d) Tryptic soy agar (TSA) (e) LB agar

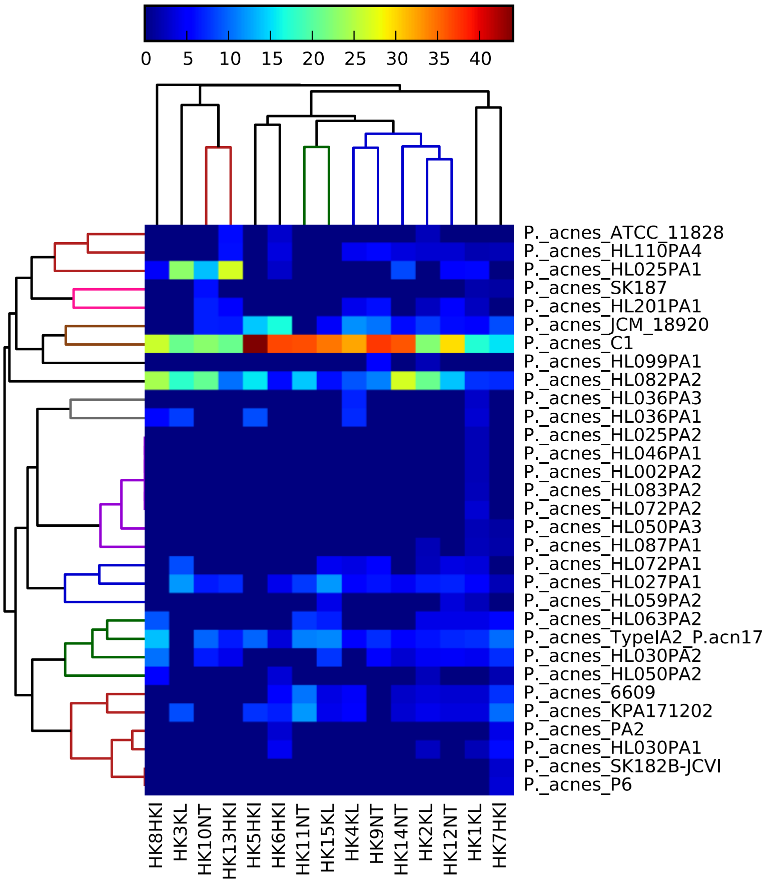

Figure S2. Heat map of P. acnes strains in Hong Kong banknotes.

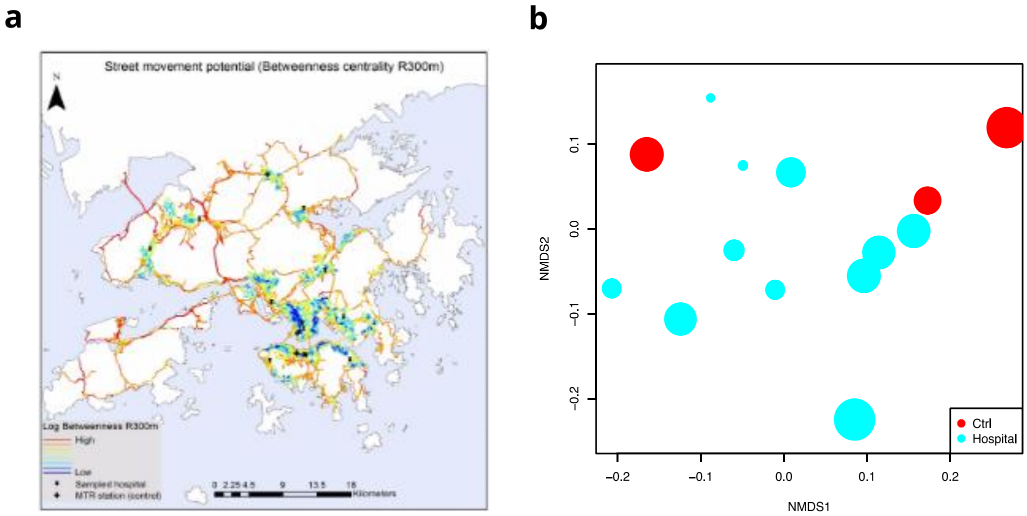

Figure S3. Correlation of betweenness centrality and microbial compositions. (a) sDNA modelled street movement potential (betweenness weighted by population density, services and street link length) at multiple spatial scales. (b) Beta-diversity (Weighted UniFrac distances) and the betweenness centrality in small-scale networks (p-value=0.0297 at 300m scale, ADONIS).

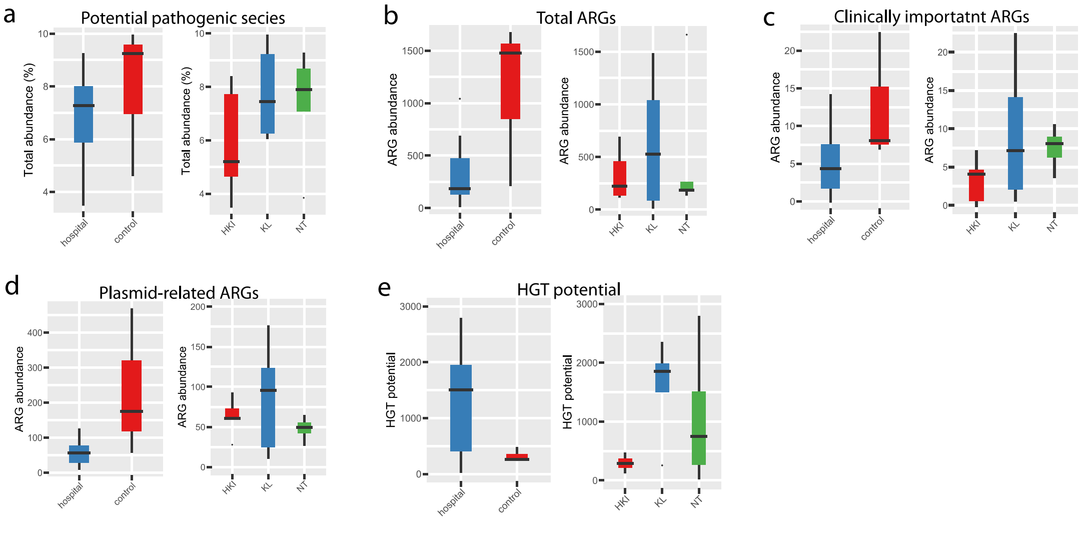

Figure S4. Comparison of relative abundance of pathogens and ARGs among all bacteria found on the HK banknotes based on metagenomic data. (a) Total abundance of pathogens. (b) Total abundance of ARG. (c) Total abundance of clinically important ARGs. (d) Total abundance of Plasmid ARGs. (e) Total HGT potential.

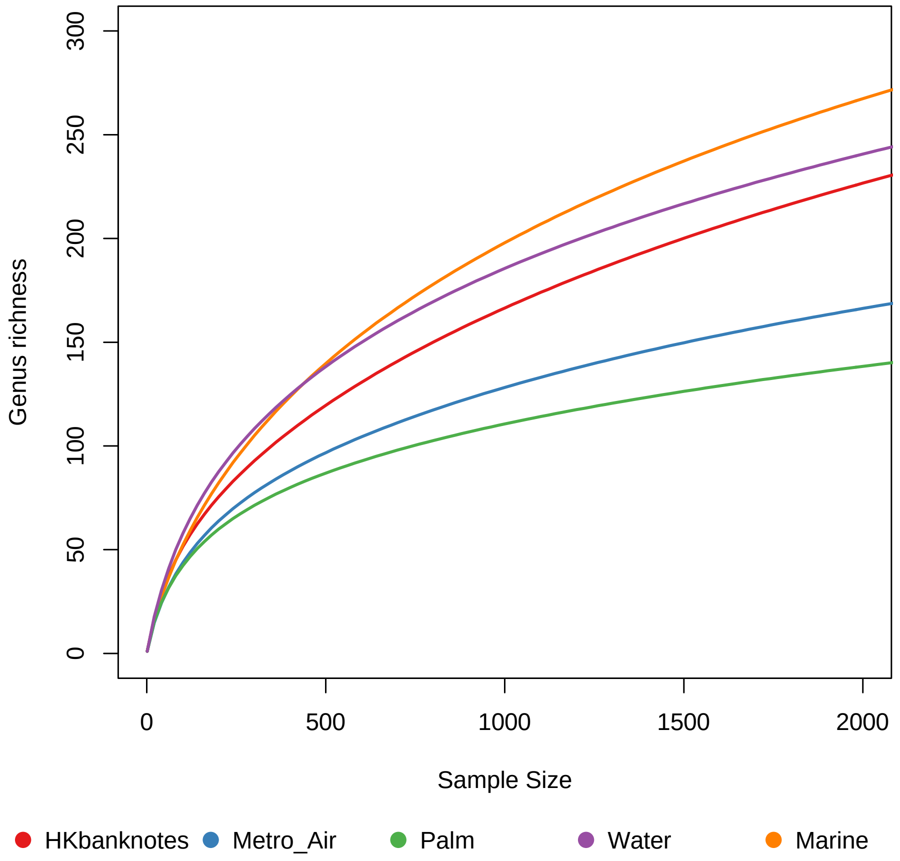

Figure S5. Comparison of genus richness among HK banknotes and other local environments.

Media	Organisms
Phenylethyl alcohol agar w/ 5% Sheep Blood	*Streptococci, Enterococci (group D), Staphylococci, Micrococci, Corynebacteria, Candida, Listeria monocytogenes, Gram-negative bacteria*
Chocolate agar	*Neisseria gonorrhoeae, Neisseria meningitidis, Streptococcus pneumoniae, Streptococcus pyogenes, Haemophilus influenzae*
Bile Esculin (B.E.) agar	*Enterococcus faecalis, Streptococcus pyogenes, Proteus mirabilis*
Tryptic soy agar (TSA)	*Escherichia coli, Staphylococcus aureus, Streptococcus pneumoniae, Streptococcus pyogenes*

Table S1. The information of selective media.

ID	Tet	Sp	Chl	Cip	Km	Ery	Amp
**HK1KL**	1	200	4	1	6	6	200
**HK2KL**	6	40	4	1	1	1	200
**HK3KL**	40	40	4	1	1	1	20
**HK4KL**	8	40	6	1	6	4	200
**HK5HKI**	1	6	2	1	1	1	1
**HK6HKI**	4	40	8	1	1	2	200
**HK7HKI**	6	40	6	1	1	20	20
**HK8HKI**	20	20	4	40	4	2	40
**HK9NT**	1	20	4	1	40	2	40
**HK10NT**	8	40	6	1	1	1	20
**HK11NT**	6	20	6	1	1	1	8
**HK12NT**	40	200	80	6	40	60	200
HK13HKI	4	8	6	1	1	1	8
HK14NT	10	40	6	2	1	1	40
HK15KL	1	20	4	1	1	1	10

Table S2. Antibiotic resistance level measured by minimum inhibitory concentration (MIC).

The sample with bolded IDs were from hospitals and italic IDs were from stations. The concentration points: 1,2,4,6,8,10,20,40,60,80,100,200. Tet: tetracycline; Sp: Spectinomycin; Chl: Chloramphenicol; Cip: Ciprofloxacin; Km: Kanamycin; Ery: Erythromycin; Amp: Ampicillin.

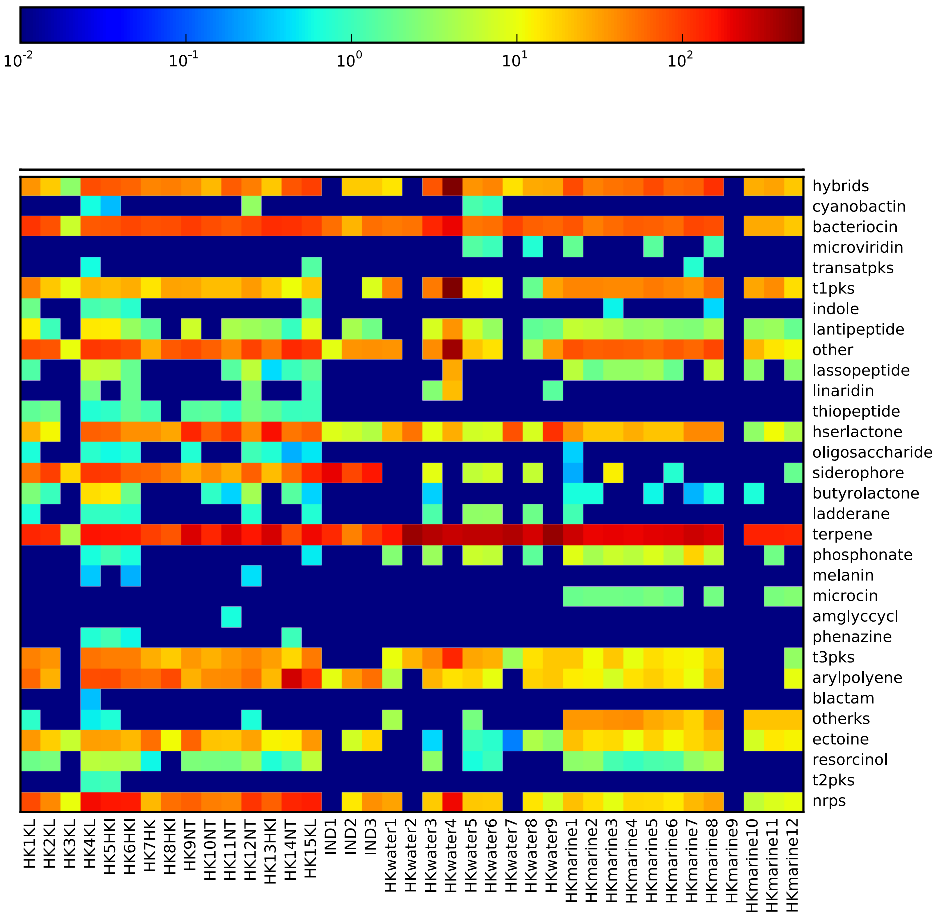

Figure S6. Heat map of biosynthetic gene clusters (BGC).

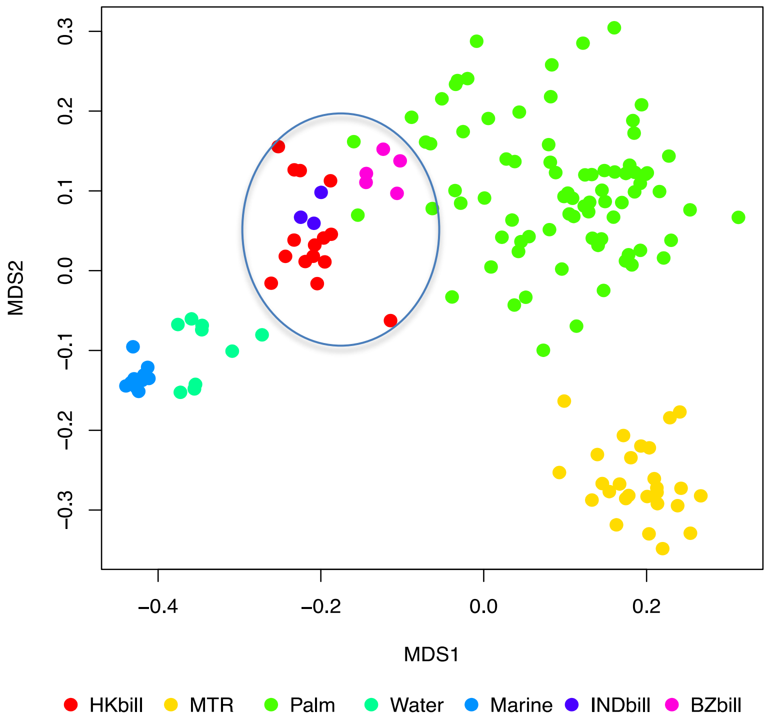

Figure S7. MDS plot for taxa comparison at 16S level using Bray-Curtis distance based on 43 genera with mean > 0.5%. The HK banknotes (red color) were distinct from other environmental samples (p-value=0.0001).

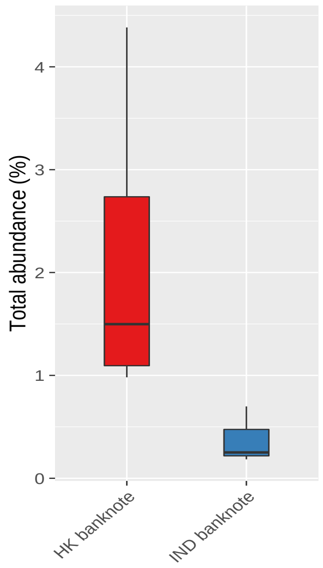

Figure S8. Comparison of marine species abundances between HK and Indian banknotes (p-value=0.002451, Wilcoxon).
